# Structural Insights into the ADCC Mechanism and Resistance of Mogamulizumab, a First-in-Class Anti-CCR4 Therapy for Cutaneous T Cell Lymphoma

**DOI:** 10.3390/ijms26125500

**Published:** 2025-06-08

**Authors:** Seung Beom Choi, Hyun Tae Lee, Nahyeon Gu, Yu-Jeong Jang, Ui Beom Park, Tae Jun Jeong, Sang Hyung Lee, Yong-Seok Heo

**Affiliations:** Department of Chemistry, Konkuk University, 120 Neungdong-ro, Gwangjin-gu, Seoul 05029, Republic of Korea

**Keywords:** mogamulizumab, antibody, C-C chemokine receptor 4 (CCR4), cutaneous T cell lymphoma (CTCL), antibody-dependent cellular cytotoxicity (ADCC), drug resistance, X-ray structure

## Abstract

Mogamulizumab is a humanized monoclonal antibody that targets C-C chemokine receptor 4 (CCR4) present on certain T cells in lymphomas and leukemias. This antibody-based therapy has demonstrated efficacy in treating various cutaneous T cell lymphomas (CTCLs), including mycosis fungoides and Sézary syndrome, through the depletion of CCR4-expressing T cells by antibody-dependent cellular cytotoxicity (ADCC). However, the precise epitope and binding mode of mogamulizumab responsible for its augmented ADCC activity remain undisclosed. Here, X-ray crystallographic studies of mogamulizumab in complex with a 28-residue N-terminal peptide indicated that SIYSNYYLYES (residues 14–24) would constitute the antibody epitope. Another high-resolution structure, using a short core peptide of these 11 residues, has elucidated unambiguous electron density for the bound peptide, confirming consistent binding for both peptides. This linear epitope is located in the membrane-proximal region of CCR4, facilitating the Fc-mediated effector functions, including ADCC. The structures also provide insights into the molecular basis for the resistance of the CCR4 L21V variant to mogamulizumab, which is due to a lack of structural complementarity with mogamulizumab binding. Understanding the structural basis for the mechanism of action of mogamulizumab is crucial for optimizing anti-CCR4 therapeutics to improve treatment outcomes for patients with these challenging diseases.

## 1. Introduction

Chemokines and their receptors promote cellular migration and localization within peripheral tissues. C-C chemokine receptor 4 (CCR4) has been identified as a skin-homing receptor usually expressed on T helper (Th2) cells and regulatory T (Treg) cells to facilitate the cell trafficking of lymphocytes to the skin [1]. In response to its chemokine ligands, CCL17 and CCL22, which are produced by monocytes, macrophages, and dendritic cells, CCR4 expression facilitates T cell migration to the skin [2]. Cutaneous T cell lymphomas (CTCLs) are a rare and incurable type of non-Hodgkin lymphomas originating from the clonal proliferation of skin-homing T cells [3]. The most prevalent subtype of CTCL is mycosis fungoides (MF), while the more aggressive and uncommon leukemic variant is Sézary syndrome (SS) [4]. Early-stage CTCL is primarily treated using skin-directed medications, whereas later-stage MF and SS require systemic therapies, including chemotherapy, retinoids, interferons, and histone deacetylase inhibitors such as vorinostat [5]. Advanced-stage CTCL remains challenging to manage due to the comparatively poor overall response rate associated with the systemic therapies [6]. Therefore, more efficacious therapies are required to improve the treatment of these disorders. 

Moganulizumab is a humanized, defucosylated immunoglobulin G1 kappa monoclonal antibody that targets CCR4, resulting in the depletion of CCR4-expressing cells through increased antibody-dependent cellular cytotoxicity (ADCC) [7]. This effector function of mogamulizumab is initiated when its Fc region interacts with FcγRIIIa on NK cells [8]. The fucose moiety within the antibody was removed via glycoengineering to enhance the binding affinity and reduce the effector–target cell ratio necessary for sufficient ADCC [9]. Alongside the depletion of malignant cells, Th2 and Treg cells are also eliminated [10]. The depletion of Treg cells may be an unintended yet advantageous outcome of mogamulizumab therapy [11]. Treg cells in the tumor microenvironment facilitate the immune evasion of cancer cells; thus, lowering the number of Treg cells via mogamulizumab therapy may indirectly inhibit the proliferation of malignant cells in the tumor microenvironment [12,13]. In 2018, the US FDA approved mogamulizumab for the treatment of CTCLs, including MF and SS [14]. In CTCL, there is a significant migration of T cells to the skin due to the overexpression of CCR4 on malignant T cells and the excessive production of CCR4 ligands by skin cells [15]. Mogamulizumab has demonstrated efficacy in CTCL by binding CCR4 and promoting the antibody-dependent cytotoxicity of the malignant T cells in these patients [14]. A Phase III clinical study demonstrated that mogamulizumab outperformed vorinostat in overall response rates and progression-free survival [16]. Despite the superior therapeutic efficacy of mogamulizumab in CTCL, resistance to the antibody drug has emerged in many patients with MF and SS, resulting in mutations involving CCR4 [17]. However, the resistance mechanisms associated with these mutations remain unexplained.

Antibodies can elicit several Fc-mediated effector functions to deplete target cells, including antibody-dependent cellular cytotoxicity (ADCC), complement-dependent cytotoxicity (CDC), and antibody-dependent cellular phagocytosis (ADCP). Which of these processes is more critical for effectiveness is primarily defined by the target molecule’s intrinsic characteristics. However, the binding of monoclonal antibodies to different epitopes within the same protein induces varying degrees of therapeutic efficacy. The distance of an antibody to the cell membrane profoundly influences its capacity to activate Fc-mediated effector functions [18]. Antibodies that attach to epitopes near the membrane are more proficient at inducing ADCC and CDC, whereas antibodies that bind to more distant epitopes are more effective at initiating ADCP.

Mogamulizumab targets the extracellular N-terminal region of CCR4 to elicit ADCC [19]. Although it has been approved and used in clinics, the detailed interaction between mogamulizumab and CCR4 remains unclear, partly because the structure of the CCR4–mogamulizumab complex has not been determined. Moreover, the structure of CCR4 itself has never been elucidated. This incomplete structural information constrains the structure-guided design of optimum anti-CCR4 agents. Here, we present the crystal structures of the N-terminal region of CCR4 in complex with mogamulizumab to elucidate its specific binding mode and precise epitope, thereby providing insights into the mechanism of action for the enhanced ADCC of mogamulizumab and the structural basis of resistance to this antibody therapy. The high-resolution structures provided in this study would also provide helpful information for the design of improved CCR4-targeting therapeutics and effective combination therapies to overcome mogamulizumab resistance for the treatment of CTCL.

## 2. Results

### 2.1. Structure of Mogamulizumab with Bound CCR4 Peptides

The Fab fragment of mogamulizumab, expressed in the periplasmic space of *E. coli*, was purified and mixed with a peptide of the CCR4 N-terminal region (residues 2–29) for crystallization. However, we failed to obtain a crystal of diffraction quality. To enhance the likelihood of crystallization, we incorporated a nanobody that can attach to the kappa light chain of a human antibody [20,21].

**Figure 1:**
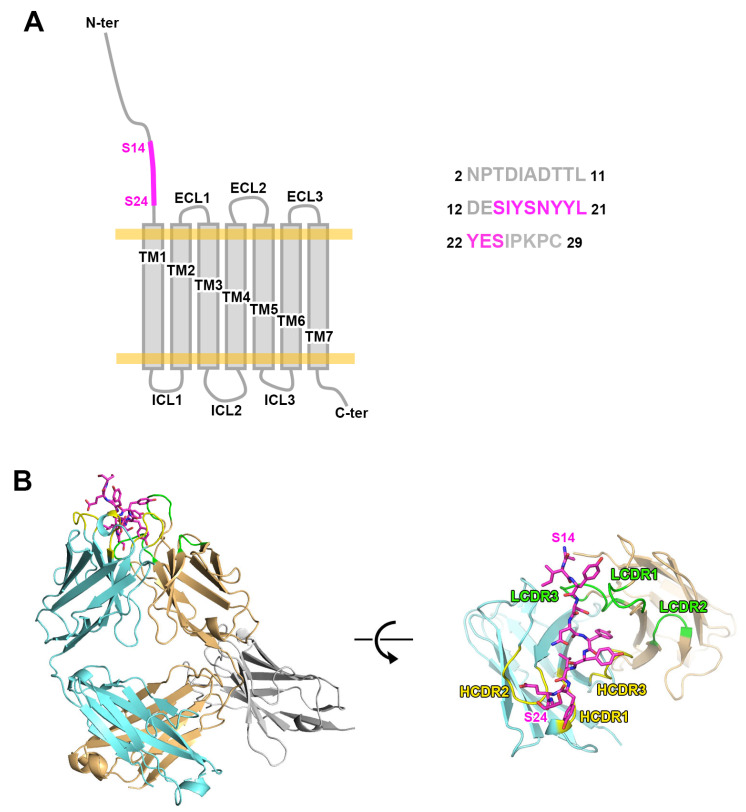
The crystal structure of mogamulizumab in complex with CCR4 N-terminal peptide. (**A**) The schematic drawing of CCR4 consisting of seven transmembrane (TM) helices, extracellular loops (ECLs), intracellular loops (ICLs), and a long N-terminal region. The amino acid sequence of the 28-residue peptide used in this study is presented. The residues S14 to S24, which were included in the structure of the ternary complex, are colored purple. (**B**) The overall structure of the ternary complex includes mogamulizumab Fab (cyan and orange), a nanobody (gray), and a 28-residue peptide (purple). The CDRs of heavy and light chains are colored yellow and green, respectively.

**Figure 2:**
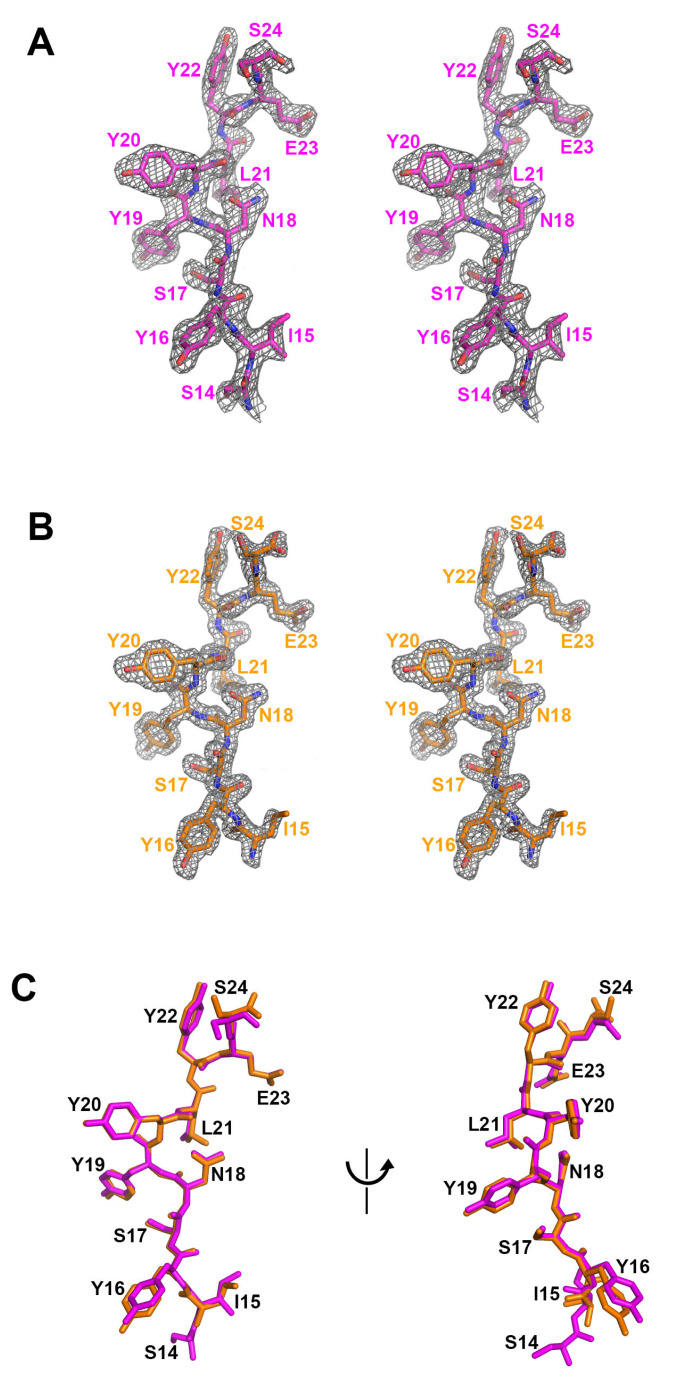
The comparison of the bound peptides. (**A**) The residues 14 to 24 of the 28-residue peptide within the ternary complex structure. A stereoscopic view of 2fofc electron density map calculated at 2.01 Å resolution and 1.2 σ contour level. (**B**) The residues 15 to 24 of the 11-residue peptide within the binary complex structure. A stereoscopic view of 2fofc electron density map calculated at 1.63 Å resolution and 1.2 σ contour level. (**C**) The superposition of the peptide residues within the ternary (purple) and binary (orange) complex structures.

The crystal structure of the mogamulizumab Fab/nanobody in complex with the 28-residue peptide was determined at a resolution of 2.01 Å, with *R/R*_free_ values of 0.197/0.244 (Figure 1). The crystals belong to the space group *P*1, with an asymmetric unit comprising two copies of the protein complex. During refinement, the extra electron density surrounding the complementarity-determining regions (CDRs) of mogamulizumab was investigated with the amino acid sequence of the 28-residue peptide. The electron density was appropriately modeled with the linear segment spanning residues S14 to S24 (Figure 2A, Supplementary Figure S1). The crystal packing allows the solvent channels to adequately accommodate the remainder of the 28-residue peptide at both ends beyond the linear segment of 11 residues. To validate the binding epitope, crystallization was attempted using the mogamulizumab Fab combined with an 11-residue peptide corresponding to the residues 14–24 of CCR4.

The crystal structure of the mogamulizumab Fab in complex with the 11-residue peptide was determined at a resolution of 1.63 Å, with *R/R*_free_ of 0.168/0.197. The crystal space group is *P*1, with two copies of the protein complex in an asymmetric unit. However, the crystal of the binary complex is entirely different from that of the ternary complex, mogamulizumab Fab/nanobody/28-residue peptide, in terms of unit cell parameters and molecular packing. In the binary complex, extra electron density also appeared in the same position as in the ternary complex. The higher resolution of the binary complex structure provided a more apparent electron density for the attached peptide, facilitating model building. The electron density encompassed the residues 15–24, implying that the first residue S14 does not participate in the interaction with mogamulizumab (Figure 2B, Supplementary Figure S2). The placement of the residues I15 to S24 in the two structures is highly analogous, confirming consistent binding for both peptides (Figure 2C). As mogamulizumab is a humanized monoclonal antibody against the N-terminal region of CCR4, this linear segment containing residues 15–24 is the epitope of the antibody drug. Given that this epitope is located proximally to the cell membrane, the ADCC activity of mogamulizumab could be augmented.

### 2.2. Interactions Between Mogamulizumab and CCR4

**Figure 3:**
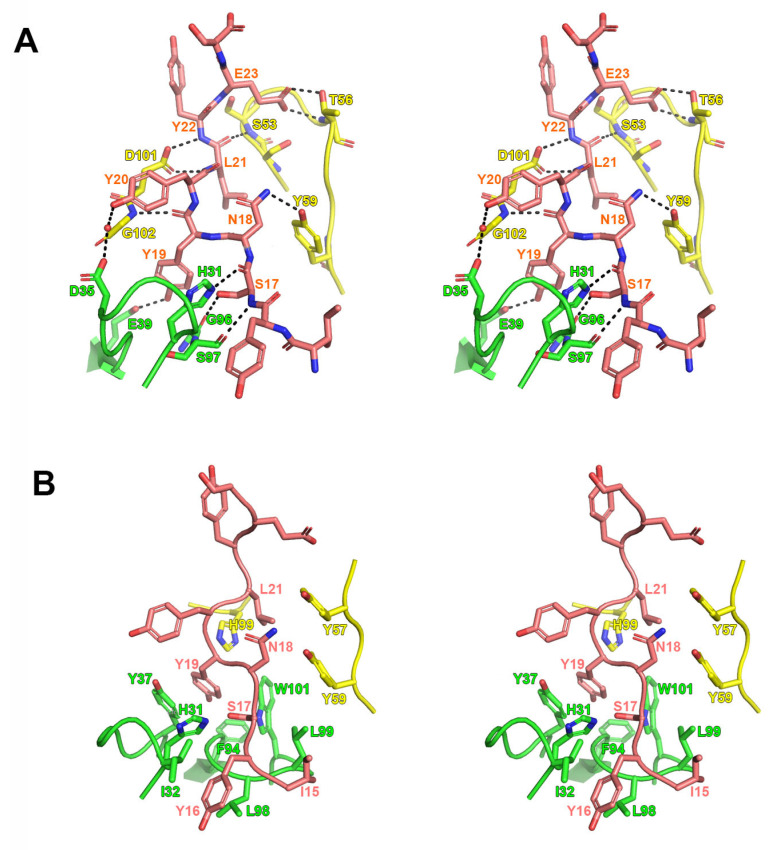
The detailed interactions within the CCR4/mogamulizumab interface. (**A**) A stereoscopic view of the hydrogen bonds between CCR4 and mogamulizumab. Hydrogen bonds are depicted as dotted lines. (**B**) A stereoscopic view of the van der Waals interactions between CCR4 and mogamulizumab. CCR4 and the heavy and light chains of mogamulizumab are colored salmon, yellow, and green, respectively.

The high resolution of the complex structures enabled the identification of the detailed interactions between CCR4 and mogamulizumab. The CCR4 binding of mogamulizumab is mediated by four complementarity-determining regions (CDRs), leaving HCDR1 and LCDR2 uninvolved. The mogamulizumab binding involves nine residues of CCR4 (residues 15–23) through hydrogen bonds and van der Waals interactions (Figure 3). The mogamulizumab residues _heavy_S53, _heavy_T56, _heavy_Y59, _heavy_D101, _heavy_G102, _light_H31, _light_E39, _light_S97, and _light_G96 form direct hydrogen bonds with the residues of CCR4, including S17, N18, Y19, L21, Y22, and E23 (Figure 3A). The high-resolution electron density could visualize a water molecule that mediates hydrogen bonds between Y20 of CCR4 and _light_D35. The side chains of the CCR4 residues, I15, Y16, S17, N18, Y19, and L21, generate significant van der Waals interactions with the side chains of the mogamulizumab residues, including _heavy_Y57, _heavy_Y59, _heavy_H99, _light_H31, _light_I32, _light_Y37, _light_F94, _light_L98, _light_L99, and _light_W101. These interactions would contribute to the specific and high affinity of mogamulizumab to CCR4, facilitating the depletion of CCR4-expressing cells via enhanced ADCC. To investigate how mogamulizumab changes when it binds to CCR4, we determined the crystal structure of the mogamulizumab Fab attached to an anti-kappa nanobody at a resolution of 2.94 Å, with *R/R*_free_ values of 0.226/0.276 (Table 1). Despite the absence of a CCR4 peptide, the electron density of mogamulizumab was clearly visible in the whole structure of the Fab fragment, containing all six CDRs. A structural comparison of the mogamulizumab CDR loops before and after binding to the N-terminal region of CCR4 revealed little conformational deviation, even in the side chains engaged in the interaction with CCR4 (Supplementary Figure S3). This result suggests that mogamulizumab maintains their CDRs in optimal conformations before interacting with CCR4, thus facilitating high-affinity binding.

### 2.3. Structural Insights into Resistance to Mogamulizumab

**Figure 4:**
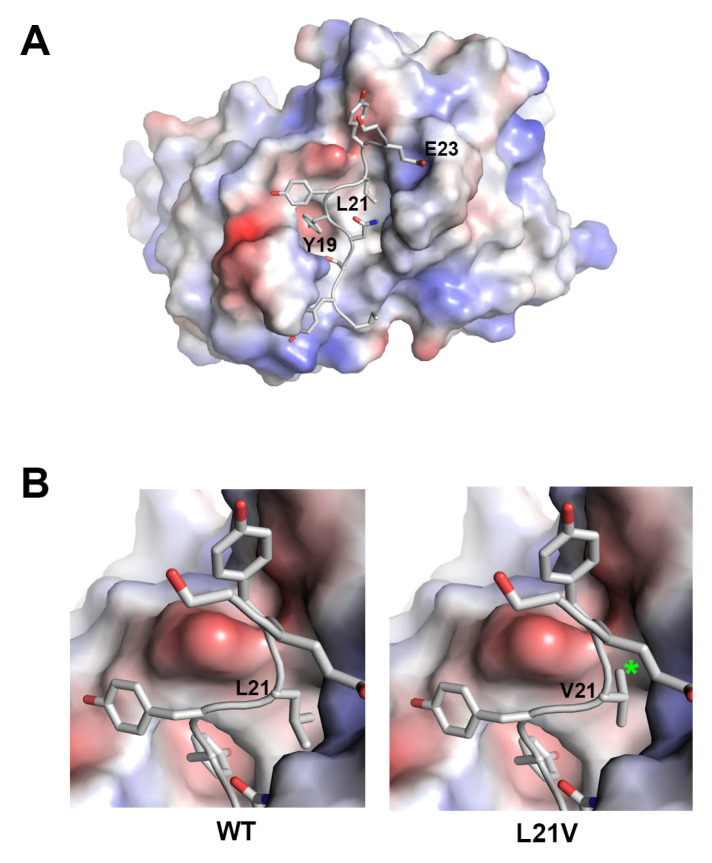
L21V mutation in resistance to mogamulizumab. (**A**) The surface representation of mogamulizumab with bound CCR4 peptide. The key residues of CCR4 for interactions are labeled. (**B**) The comparison of the interaction in the residue at position 21 between WT and L21V variant CCR4. The asterisk indicates a steric collision by the β-branched methyl group in the mutated valine residue. The surface is colored by electrostatic potential.

Despite the superior clinical results of mogamulizumab in CLTL, many patients eventually develop resistance to this antibody drug [22]. The loss of CCR4 expression is a common mechanism of resistance that explains why mogamulizumab does not work for CLTL patients. A recent study has shown that the mutation L21V in CCR4 is associated with resistance to mogamulizumab [17]. Since the mutation is positioned within the mogamulizumab-binding epitope in CCR4, the high-resolution structure from this study helps us understand how the resistance to mogamulizumab works. In the structure of mogamulizumab in complex with CCR4 peptide, the CCR4 residues, Y19, L21, and E23, play key roles in the interaction with mogamulizumab by occupying the central pockets surrounded by the CDRs of mogamulizumab (Figure 4A). The mutation, L21V, can affect the binding affinity of the CCR4 variant to mogamulizumab. The mutated residue in the CCR4 L21V variant cannot neatly occupy the pocket due to the smaller size of valine compared to leucine (Figure 4B). Furthermore, the branched methyl group on the β position of the mutated valine residue can sterically collide with the surface of the pocket, thereby messing up the core interactions mediated by the residues Y19, L21, and E23. Antibody design based on the structure of the mogamulizumab/CCR4 complex may yield a novel anti-CCR4 antibody that can evade this mutational resistance to mogamulizumab.

### 2.4. Molecular Mechanism of Mogamulizumab ADCC

**Figure 5:**
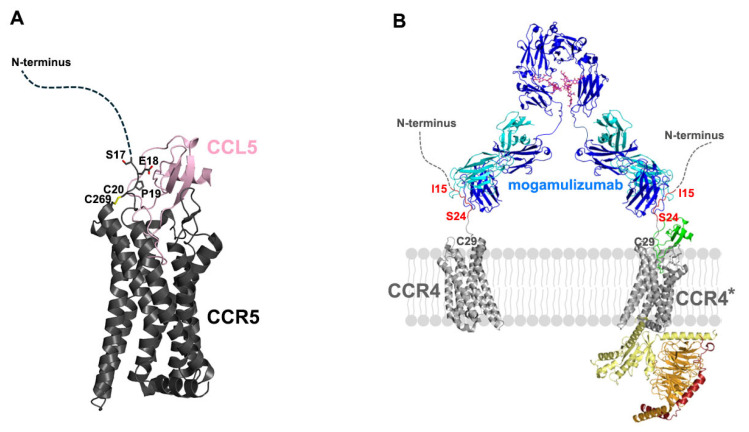
The molecular mechanism of mogamulizumab ADCC. (**A**) The structure of CCR5 in complex with its ligand CCL5 (PDB entry: 7O7F). The disulfide bond between C20 and C269 is depicted. The residues E18 and P19, which are involved in ligand binding, are labeled. The flexible N-terminal region is indicated by a dotted curve. (**B**) The hypothetical model of the molecular architecture of mogamulizumab binding. The two variable regions of mogamulizumab can bind to the epitope (red, residues 15–24) in both inactive CCR4 and active CCR4 (CCR4*). The heavy and light chains of mogamulizumab are colored blue and cyan. The glycans within Fc are depicted as a purple stick model. The ligand of CCR4 is colored green, and the G protein subunits are colored yellow, orange, and red.

Despite the absence of the structure of CCR4 or its complexes with binding partners, including ligands or antibodies, the complete architecture of the mogamulizumab-binding CCR4 can be hypothesized from the structures of other homologous class A GPCRs, including CCR1, CCR2, CCR3, CCR5, CCR6, CCR8, and CCR9 [23,24,25,26,27,28]. In class A GPCRs, the cysteine residue at position 29 of CCR4 is fully conserved and located at the membrane surface, linked by a disulfide bond to another cysteine residue in a transmembrane helix of GPCRs. The human C-C chemokine receptor 5 (CCR5) exhibits 50% identity in its amino acid sequence with CCR4, and its molecular structures for ligand recognition and activation are well characterized. CCR5 also has a long N-terminal region, as does CCR4, with the residue C20 making a disulfide bond to C269 [25]. In its active form complexed with its ligand CCL5, only the two residues adjacent to C20, P19 and E18 are involved in the interaction with the ligand. In contrast, the prior N-terminal residues (residues 1–17) have been missing from the structure, suggesting their non-involvement in ligand binding (Figure 5A). The mogamulizumab epitope (residues 15–24) is positioned four residues away from the conserved cysteine, C29 of CCR4, and an AlphaFold3-predicted model demonstrates that the N-terminal region of CCR4 does not participate in ligand binding except that P28 of CCR4 interacts with CCL17 through van der Waals contacts (Supplementary Figure S4) [29]. This suggests that mogamulizumab binding does not interfere with ligand binding to CCR4. Therefore, mogamulizumab can bind to the membrane-proximal epitope in CCR4, regardless of whether it is in its inactive free form or its active form with a bound ligand for intracellular signaling (Figure 5B). Mogamulizumab’s binding is not contingent upon the presence of the ligand bound to CCR4, perhaps resulting in a more enhanced ADCC through binding to both types of CCR4, unlike antibodies that target only one type.

## 3. Discussion

Mogamulizumab is a defucosylated IgG1 kappa monoclonal antibody that depletes CCR4-expressing cells through potent ADCC for the treatment of CTCL. However, some patients exhibited resistance to mogamulizumab, with disease relapse occurring around 14 months in responders [22]. Mutations associated with resistance in CCR4 have been identified, indicating that the L21V variant affects the N-terminal region containing a putative epitope of mogamulizumab, whereas the M116R and T161-W162 delinsSR mutations, located within the transmembrane domain of CCR4, are correlated with the loss of CCR4 protein expression [17]. The structure presented in this study provides insights into the molecular basis of mogamulizumab resistance associated with the CCR4 L21V mutation, offering information for the development of more effective anti-CCR4 therapies and combination regimens to address this resistance.

Nowdays, monoclonal antibodies are a major therapeutic approach due to their exceptional specificity and affinity for targets. Identifying the specific epitope of an antibody medication is essential for understanding the mechanism underlying its therapeutic efficacy, as different epitopes may yield varying therapeutic outcomes for antibodies. Several investigations have shown that the distance of antibodies from the cell membrane regulates Fc-mediated effector functions, including CDC, ADCC, and ADCP. Decreasing the distance between the antibody-binding epitope within CD33 and the leukemia cell membrane by around 4 nm was sufficient to induce changes in the ADCC activity [30]. Furthermore, targeting the membrane-proximal domain of CD33 has yielded enhanced efficacy, underscoring the advantages of membrane-proximal targeting of CD33 for the effectiveness of chimeric antigen receptor-natural killer (CAR-NK) cells [31]. Trastuzumab, an anti-HER2 antibody targeting a membrane-proximal epitope, elicits superior ADCC compared to pertuzumab, which binds to a membrane-distal epitope within HER2, suggesting that the epitope’s characteristics can affect Fc-mediated effector functions [32]. Ofatumumab, an anti-CD20 antibody that binds to a membrane-proximal loop epitope, also induces higher levels of ADCC than rituximab [33]. Moreover, it has been observed that targeting membrane-proximal epitopes is advantageous for CAR-T therapy. A reduction in CAR-T cytotoxicity was seen when the binding epitopes of CAR were placed further from the target cell membrane [34].

This study elucidates the crystal structure of mogamulizumab in complex with CCR4, demonstrating that the antibody binds to the membrane-proximal linear epitope in the N-terminal region of CCR4, thereby inducing potent ADCC that underpins the therapeutic efficacy of the antibody by depleting CCR4-expressing cells. This structural analysis of the hotspots on CCR4 may facilitate the development of more effective anti-CCR4 therapeutics. In addition, the insights gained from the complex structure of mogamulizumab and its epitope peptide may offer critical information for designing a novel cancer vaccine that elicits an immune response against CCR4 for the treatment of CTCL. Investigative studies of off-target effects induced by monoclonal antibodies in large cohorts have demonstrated that target specificity is not as high as previously assumed, raising new concerns regarding the polyreactivity and polyspecificity of antibodies [35,36]. The accumulation of structural studies regarding the recognition of diverse targets by antibodies may help enhance the target specificity of therapeutic antibodies.

## 4. Materials and Methods

### 4.1. Protein Expression and Peptide Preparation

Supplementary Materials provide details on the expression and purification of the mogamulizumab Fab fragment and the anti-kappa nanobody. In short, a modified pBAD vector was used to insert the DNA sequences for the heavy and light chains of the mogamulizumab Fab, which were synthesized after codon optimization for *E. coli* expression. Ni-affinity and gel filtration chromatography were employed for the purification of the mogamulizumab Fab, which was expressed in the periplasmic space of *E. coli*. The DNA sequence for the nanobody was synthesized after codon optimization for *E. coli* expression and subsequently inserted into the pET21b expression vector (Merck Millipore, Burlington, MA, USA) [20]. The nanobody protein was expressed in *E. coli* BL21(DE3) cells as inclusion bodies and subsequently refolded into a soluble form. Ni-affinity and gel filtration chromatography were employed to purify the nanobody. The peptides were synthesized by Dandi Cure (Ochang, Korea): NPTDIADTTLDESIYSNYYLYESIPKPC (28 residues) and SIYSNYYLYES (11 residues).

### 4.2. Crystallization of Proteins

For the crystallization of the ternary complex, mogamulizumab Fab/nanobody/28-residue peptide, mogamulizumab’s Fab fragment was mixed with the nanobody and 28-residue peptide at a molar ratio of 1:1.2:2, followed by a 30 min incubation on ice. The mixture was loaded on a HiLoad 26/600 Superdex 200 pg column (Cytiva, Marlborough, MA, USA) for gel filtration chromatography with a buffer containing 20 mM Tris, pH 7.5, and 200 mM NaCl. The purified ternary complex was concentrated to 10 mg mL^−1^ and crystallized via hanging-drop vapor diffusion with a reservoir solution of 0.1 M sodium HEPES pH 6.5, 20% (*w*/*v*), and PEG 10,000 at 20 °C. Before the X-ray diffraction experiment, crystals were immersed in the crystallization reservoir solution containing an additional amount of 20% (*v*/*v*) ethylene glycol and rapidly frozen in liquid nitrogen. For the crystallization of the binary complex, mogamulizumab Fab/11-residue peptide, mogamulizumab’s Fab fragment was mixed with the 11-residue peptide at a molar ratio of 1:2, and the mixture was further purified by gel filtration chromatography with the same buffer condition. The purified binary complex was concentrated to 14 mg mL^−1^ and crystallized by hanging-drop vapor diffusion with a well solution containing 0.1 M sodium HEPES pH 7.5, 20% (*w*/*v*) PEG 4000, and 10% (*v*/*v*) 2-propanol at 20 °C. Prior to the X-ray diffraction experiment, crystals were soaked in the crystallization reservoir solution plus 20% (*v*/*v*) glycerol and quickly frozen in liquid nitrogen. To crystallize the complex of mogamulizumab Fab and the anti-kappa nanobody, the purified Fab fragment and nanobody were mixed in a molar ratio of 1:1.2, and the extra nanobody protein was removed by size exclusion chromatography. The purified protein of the complex was concentrated to 12 mg mL^−1^ and applied to crystallization through the hanging-drop method using a reservoir solution containing 0.02 M DL-glutamic acid, 0.02 M DL-alanine, 0.02 M glycine, 0.02 M DL-lysine monohydrochloride, 0.02 M DL-serine, 0.1 M sodium HEPES, MOPS(acid) pH 7.5, 12.5% (*v*/*v*) MPD, 12.5% (*w*/*v*) PEG 1000, and 12.5% (*w*/*v*) PEG 3350 at 20 °C. The crystals were frozen in liquid nitrogen without any additional cryoprotectant.

### 4.3. Data Collection and Structure Determination

The X-ray diffraction data of the protein crystals were collected at Pohang Accelerator Laboratory (PAL) beamlines 5C and 7A, and subsequently processed and scaled automatically using XDS [37]. Molecular replacement was conducted with Phaser using the complex structure of cobolimab Fab/anti-kappa nanobody (PDB entry: 8GSI) as an initial model [20,38]. During molecular replacement, we separated the structures of the variable and constant regions of the Fab fragment and nanobody from the model structure. Model building and structure refinement were executed using PHENIX (version 1.21) and Coot (version 0.9.8.95) [39,40]. Table 1 presents the statistics for data collection and structural refinement. All structural figures were generated using PyMol (Schrödinger, Inc., Cambridge, MA, USA). The atomic coordinates and structure factors for the ternary complex, binary complex, and mogamulizumab Fab complexed with the nanobody are deposited in Protein Data Bank (http://www.rcsb.org (accessed on 20 April 2025)) under the accession codes 9ULM, 9ULL, and 9ULN, respectively.

## Figures and Tables

**Table 1:** Data collection and refinement statistics.

	Mogamulizumab /CCR4 (N2-C29) /VHH	Mogamulizumab /CCR4 (S14-S24)	Mogamulizumab /VHH
Data Collection			
X-ray Source	PLS 5C	PLS 5C	PLS 7A
Wavelength (Å)	1.0000	1.0000	1.0000
Space Group	P1	P1	P3_2_21
a, b, c (Å)	65.39, 71.19, 73.45	41.27, 67.44, 87.83	110.02, 110.02, 120.97
α, β, γ (°)	81.24, 61.41, 71.05	87.2, 82.7, 79.2	90.00, 90.00, 120.00
Resolution (Å)	2.01 (2.07–2.01) ^1^	1.63 (1.69–1.63)	2.94 (3.00–2.94)
R_meas_ (%)	9.50 (57.70)	5.40 (67.90)	7.30 (65.5)
I/σI	7.27 (1.62)	11.28 (1.59)	22.7 (2.31)
Completeness (%)	93.6 (94.6)	94.8 (93.5)	99.8 (99.6)
Redundancy	1.8 (1.9)	2.0 (1.8)	9.6 (9.7)
CC_1/2_	0.995 (0.792)	0.999 (0.710)	0.983 (0.853)
Refinement			
Resolution (Å)	2.01	1.63	2.94
No. of Reflections	70,477	109,223	17,972
R_work_/R_free_ (%)	19.7/24.4	16.8/19.7	22.6/27.6
No. of Atoms			
Protein	8481	6776	4254
Water	874	1187	0
Average B-factor (Å^2^)	37.0	27.0	71.0
R.m.s. Deviation			
Bond Lengths (Å)	0.007	0.005	0.009
Bond Angles (°)	0.885	0.765	1.292
Ramachandran			
Favored (%)	99.26	98.15	99.27
Allowed (%)	0.74	1.85	0.73
Outlier (%)	0.00	0.00	0.00
PDB Code	9ULM	9ULL	9ULN

^1^ Values in parentheses are for the outer resolution shell.

## Data Availability

The atomic coordinates and structure factors for the structures in this study were deposited in Protein Data Bank (http://www.rcsb.org (accessed on 20 April 2025)) under the accession codes 9ULM, 9ULL, and 9ULN.

## Supplementary Materials

The supporting information can be downloaded at: https://www.mdpi.com/article/10.3390/ijms26125500/s1.
