# Supplementary material for: Structural Insights into the ADCC Mechanism and Resistance of Mogamulizumab, a First-in-Class Anti-CCR4 Therapy for Cutaneous T Cell Lymphoma
Source: Int J Mol Sci. 2025 Jun 8;26(12):5500. doi: 10.3390/ijms26125500 (PMC12193575; doi:10.3390/ijms26125500)
Supplement: Supplementary file 1 [file ijms-26-05500-s001.zip › ijms-3663076-supplementary.pdf]

SUPPLEMENTARY MATERIALS

Structural Insights into the ADCC Mechanism and Resistance of Mogamulizumab, a First-in-Class Anti-CCR4 Therapy for Cutaneous T-cell Lymphoma

Seung Beom Choi†, Hyun Tae Lee†, Nahyeon Gu, Yu-Jeong Jang, Ui Beom Park, Tae Jun Jeong, Sang Hyung Lee and Yong-Seok Heo*

Expression and Purification of Mogamulizumab Fab

The DNA sequence for the Fab fragment of mogamulizumab was synthesized after codon-optimization for expression in *E. coli*. The sequences for heavy chain and light chain were cloned into a modified pBAD vector, containing the STII signal sequence in each chain for periplasmic secretion and a C-terminal 6His-tag in heavy chain. The plasmid pBAD-Fab was transformed into *E. coli* Top10F. The cells were grown at 37 ˚C in LB medium supplemented with 50 μg mL^{-1} ampicillin. At an OD_{600} of 1.0, the protein expression was induced with 0.2% arabinose and cells were grown at 30 °C for 15 h. The cells were harvested by centrifugation, re-suspended in lysis buffer (20 mM Tris, pH 8.0, 200 mM NaCl) and lysed by sonication on ice. After removing cell debris by centrifugation (25,000 × g for 0.5 h at 4°C), the supernatant containing soluble protein was applied to HisTrap HP column and washed with five column volumes of wash buffer (20 mM Tris, pH 8.0, 300 mM NaCl, 50 mM imidazole). The protein was then eluted with elution buffer (20 mM Tris, pH 8.0, 300 mM NaCl, 400 mM imidazole). The eluted protein was concentrated for gel filtration chromatography using a HiLoad 16/60 Superdex 200 pg column. The column had previously been equilibrated with gel filtration buffer (20 mM Tris, pH 8.0, 300 mM NaCl).

Expression and Purification of Anti-kappa Nanobody

DNA sequence for the nanobody wsa synthesized and subcloned into pET21b expression vector. The protein for the nanobody was expressed in *E. coli* as inclusion bodies and refolded to soluble forms. *E. coli* BL21 (DE3) cells were transformed with the plasmids described above and were grown at 37°C in LB medium supplemented with 50 μg ml^{-1} ampicillin to an OD600 of 0.6–0.8 before being induced with 0.5 mM IPTG. The cells were cultured for an extra 4 h at

37°C. Harvested cells were resuspended in a buffer containing 20 mM Tris (pH 8.0) and 200 mM NaCl, and sonicated. Buffer containing 20 mM Tris (pH 8.0), 1.5% Triton X-100, and 200 mM NaCl was added to the lysed cells. Inclusion bodies were collected by centrifugation (25,000 × g for 0.5 h at 4°C) and then dissolved in a solution containing 20 mM Tris (pH 8.0), 200 mM NaCl, 6 M guanidine hydrochloride, and 50 mM DTT by stirring at room temperature overnight. The solubilized protein was refolded by repetitive dialysis using a buffer containing 20 mM Tris (pH 8.0), 200 mM NaCl, 3 mM reduced glutathione, 1 mM oxidized glutathione, and 0.1 M arginine at 4°C. After the precipitated protein was removed by centrifugation and filtration, the refolded protein was applied to HisTrap HP column (Cytiva) and eluted with a buffer containing 20 mM Tris (pH 8.0), 200 mM NaCl, and 400 mM imidazole. The eluted protein was purified further by gel filtration chromatography using HiLoad 26/600 Superdex 200 pg column (Cytiva) with a buffer containing 20 mM Tris (pH 8.0) and 200 mM NaCl.

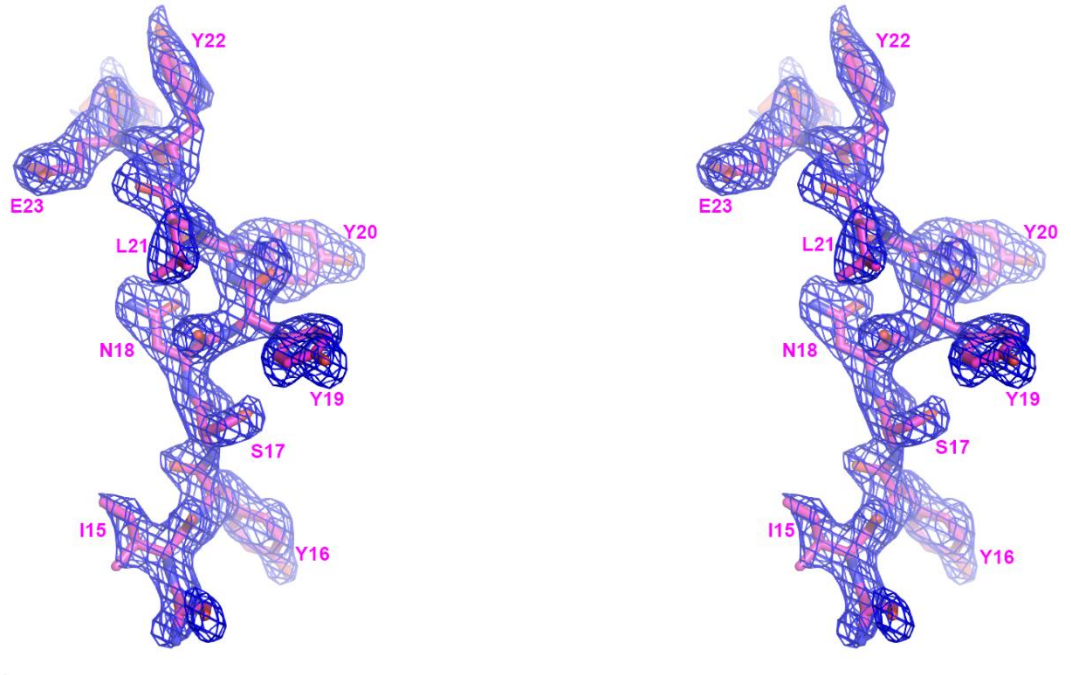

Supplementary Figure S1. Electron density on the 28-residue peptide.

A stereoscopic view of fofc map calculated at 2.01 Å resolution and 2.5 σ contour level. The residues represented as a stick model were omitted during the map calculation.

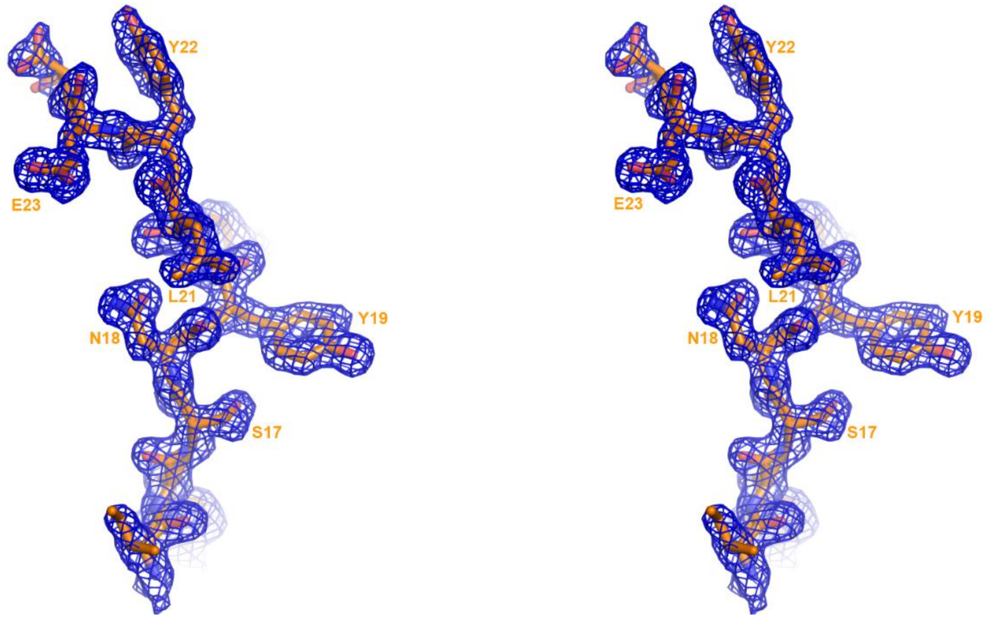

Supplementary Figure S2. Electron density on the 11-residue peptide.

A stereoscopic view of fofc map calculated at 1.63 Å resolution and 2.5 σ contour level. The residues represented as a stick model were omitted during the map calculation.

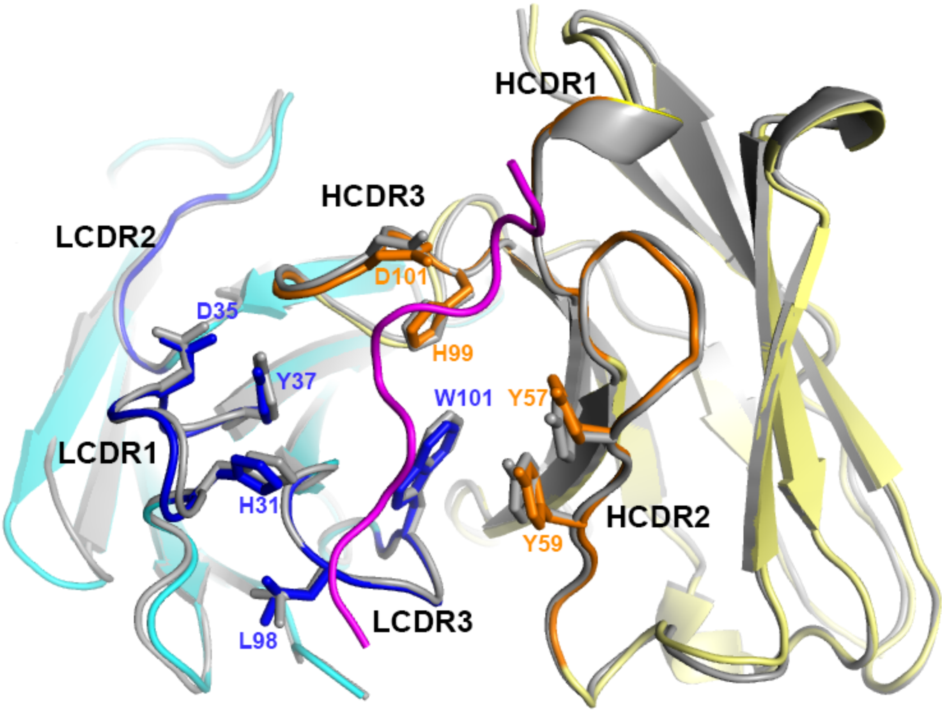

Supplementary Figure S3. Comparison of mogamulizumab structures before and after binding to CCR4 peptide.

A structural comparison of the mogamulizumab CDR loops before (gray) and after (orange and blue) binding to the N-terminal region of CCR4 showed little conformational deviation. The key residues within the mogamulizumab CDRs for the interaction with the CCR4 (purple) are labelled.

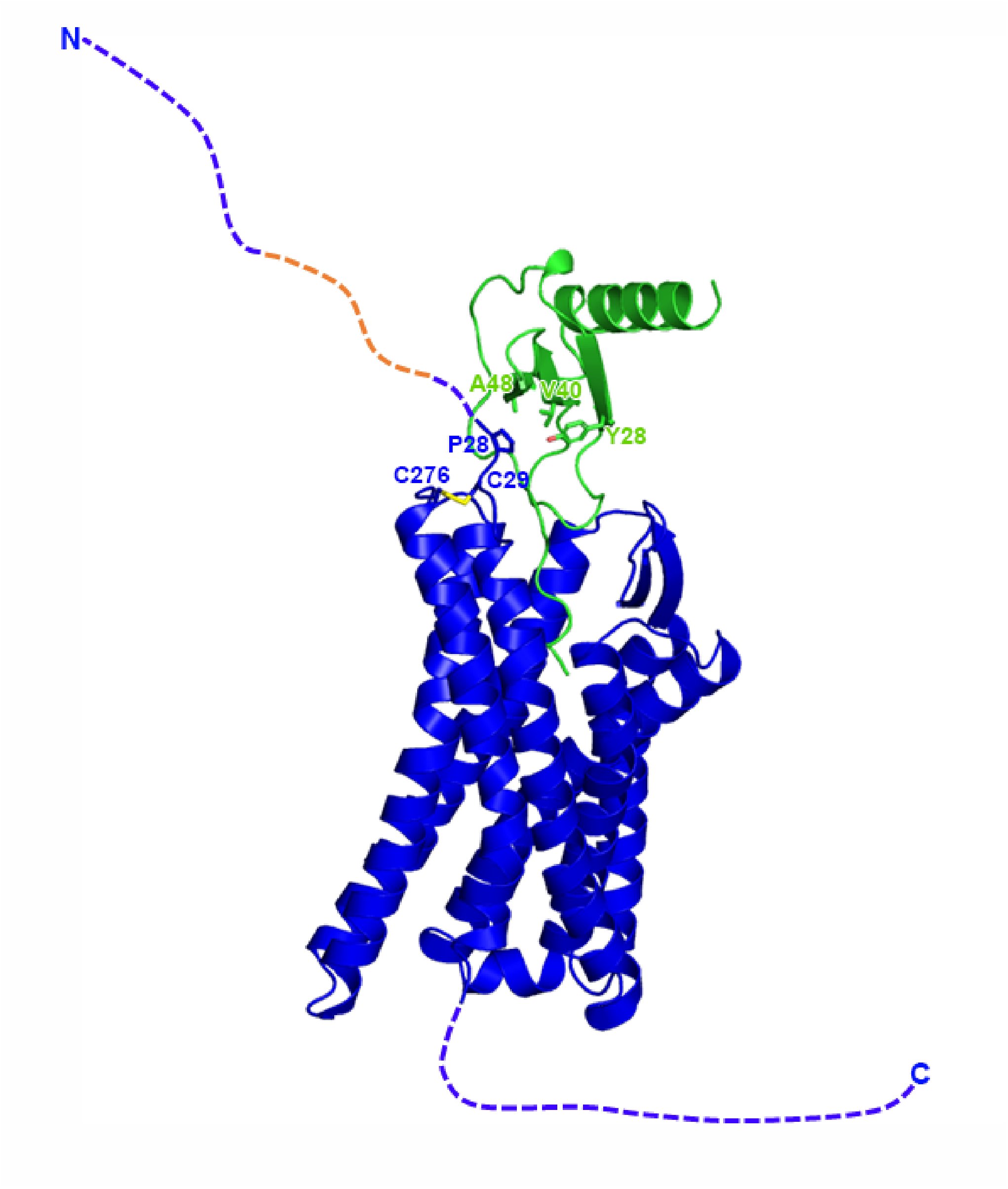

Supplementary Figure S4. AlphaFold3-predicted model of the CCR4/CCL17 complex.

Unpredicted regions of CCR4 (blue) are represented as dotted curves. The mogamulizumab epitope region is colored orange. The disulfide bond between C29 and C276 within CCR4 is represented by a yellow stick. The residues of CCL17 (green) , which interact with P28 of CCR4, are represented as a stick model.
